# Metal-induced neurodegeneration in *C. elegans*

**DOI:** 10.3389/fnagi.2013.00018

**Published:** 2013-05-20

**Authors:** Pan Chen, Ebany J. Martinez-Finley, Julia Bornhorst, Sudipta Chakraborty, Michael Aschner

**Affiliations:** ^1^Department of Pediatrics, Vanderbilt University Medical CenterNashville, TN, USA; ^2^Department of Pharmacology, the Kennedy Center for Research on Human Development, and the Center for Molecular Toxicology, Vanderbilt University Medical CenterNashville, TN, USA

**Keywords:** neurodegeneration, behavior, metals, *C. elegans*, neurotransmitters

## Abstract

The model species, *Caenorhabditis elegans*, has been used as a tool to probe for mechanisms underlying numerous neurodegenerative diseases. This use has been exploited to study neurodegeneration induced by metals. The allure of the nematode comes from the ease of genetic manipulation, the ability to fluorescently label neuronal subtypes, and the relative simplicity of the nervous system. Notably, *C. elegans* have approximately 60–80% of human genes and contain genes involved in metal homeostasis and transport, allowing for the study of metal-induced degeneration in the nematode. This review discusses methods to assess degeneration as well as outlines techniques for genetic manipulation and presents a comprehensive survey of the existing literature on metal-induced degeneration studies in the worm.

## Introduction

The simplicity of the small nematode *Caenorhabditis elegans* (*C. elegans*) makes it a suitable model organism for biomedical research. Compared with mammalian models, it has a number of advantages that makes it a powerful tool for studies on human disease. First, 60–80% of human genes have corresponding genes in *C. elegans*, depending on the specific bioinformatic methods used (Titus and Michael, 2006). Many genetic factors causing human diseases have corresponding worm homologs, allowing for mechanism-based studies. Second, the small size and the fast life-cycle of the worm allow for easy maintenance. The adult worm is only 1.3 mm long and it takes 3 days for an embryo to reach adulthood and reproduce. A 100 mm petri-dish can accommodate thousands of self-fertilizing nematodes within couple of days. This is particularly important for pharmaceutical drug screens. Third, *C. elegans* are transparent, which allows *in vivo* study with fluorescent reporters, such as green and red fluorescent proteins. This has been widely used to study axon guidance, neurodegeneration, endocytosis and fat metabolism in living worms. Fourth, the simple, but complete nervous system makes it easy to investigate neurological function in *C. elegans*. Although a worm has only 302 neurons, it shares similar neurotransmitters with humans, including dopamine (DA), acetylcholine (ACh), serotonin (5-HT), *gamma*-aminobutyric acid (GABA), glutamate, and others. Fifth, a variety of tools are available in *C. elegans*. The genome and cell line lineage have been completely mapped, RNA interference (RNAi) libraries are able to knock down 90% of genes, a large pool of knockout mutants are available from the *C. elegans* genetic center (CGC) and commercial worm-specific microarray probes have been developed.

A big advantage afforded by *C. elegans* as a model for studies on human neurological diseases is the simplicity of its nervous system: the worm contains 302 neurons and about 5,000 synapses (White et al., 1986). Despite this simplicity, *C. elegans* has a complete nervous system with four functional categories of neurons based on their circuitry: (1) motor neurons, which pass synaptic signals to muscle cells; (2) sensory neurons, which convert environmental signals into internal stimuli; (3) interneurons, which receive and deliver signals between neurons; and (4) polymodal neurons, which have two or more of the functions described above. Most commonly, studies are performed on different types of neurons based on their neurotransmitter profile.

The biosynthesis and transport of neurotransmitters are conserved in the nematode and human nervous system. Among different types of neurons, dopaminergic (DAergic) and gamma-aminobutyric acid (GABA-ergic) neurodegeneration are the two best characterized. DAergic neuron death is a well-known feature of Parkinson's disease (PD). *C. elegans* hermaphrodites have eight DAergic neurons; two pairs of cephalic (CEP) neurons, a pair of anterior deirid (ADE) neurons, and a pair of postdeirid (PDE) neurons (WormAtlas, 2002, 2003, 2004, 2005, 2006, 2007, 2008, 2009; Toth et al., 2012; WormBase, 2013). Since their genome has been fully characterized it is also known that they contain all genes responsible for DA biosynthesis, packaging and reuptake (Sulston et al., 1975; Jayanthi et al., 1998). Male *C. elegans* have additional DAergic neurons (Sulston et al., 1975). DA signaling is important in mediating learning and locomotion behavior (Sawin et al., 2000). DA is also a requirement for olfactory adaptation (Colbert and Bargmann, 1995). The role of DA in modulation of locomotor activity has been exploited to study DAergic degeneration. Using green fluorescent protein (GFP) (Nass et al., 2002; Cao et al., 2005) or mCherry protein (Harrington et al., 2012) to visualize DAergic neurons in *C. elegans*, researchers are able to monitor neurodegenerative processes in living animals. Many known causes of PD also result in DAergic neurodegeneration in *C. elegans*, including aberrant proteins [alpha-synuclein (Cao et al., 2005; Harrington et al., 2012)], heavy metals [i.e., manganese (Mn) (Benedetto et al., 2010), methylmercury (MeHg) (VanDuyn et al., 2010), aluminum (VanDuyn et al., 2013)], and environmental contaminants [i.e., fungicides (Harrison Brody et al., 2013), pesticides (Negga et al., 2012), insecticides (Mocko et al., 2010), 1-methyl-4-phenylpyridinium (MPP^+^) (Pu and Weidong, 2008; Mocko et al., 2010), and 6-hydroxydopamine (6-OHDA) (Cao et al., 2005; Ruan et al., 2010)]. The DAergic neurons are mechanosensory and defects or loss of these neurons results in behavioral changes in response to environmental stimuli (i.e., food sensing).

In vertebrates, GABA is the most abundant neurotransmitter in inhibitory synapses in the central nervous system (CNS), while in nematodes, GABA is used as both an inhibitory and excitatory neurotransmitter primarily at neuromuscular synapses, as worms do not have a CNS. GABAergic neurons in the nematode consist of 26 neurons that are categorized under six classes, DD, VD, RME, RIS, AVL, and DVB, based on their synaptic outputs. DD and VD innervate the dorsal and ventral body muscles, RME innervate the head and AVL and DVB innervate the enteric muscles and RIS are interneurons (White et al., 1986). Worms defective in GABAergic function exhibit “shrinking” behavior, whereby the nematode simultaneously hypercontracts both ventral and dorsal body muscles, as well as abnormal defecation and foraging. *unc-25* encodes the GABA enzyme glutamic acid decarboxylase and *unc-47* encodes the GABA vesicular transporter (McIntire et al., 1997; Jin et al., 1999). *unc-30* encodes a homeodomain protein of the Pitx family and regulates the differentiation of the D-type GABAergic neurons (Jin et al., 1994). Overexpression of genetic risk factors associated with Alzheimer's disease (AD) [tau (Kraemer et al., 2003; Miyasaka et al., 2005; Brandt et al., 2009)] and Amyotrophic lateral sclerosis (ALS) [TDP-43 and FUS (Vaccaro et al., 2012)], as well as exposure to several heavy metals [lead (Pb), mercury (Hg), copper (Cu), chromium (Cr), and Mn (Du and Wang, 2009; Xing et al., 2009b)] results in GABAergic neurodegeneration in nematodes. The loss or injury of GABAergic neurons alters locomotion (McLntire et al., 1993), foraging (White et al., 1986) and defecation (McLntire et al., 1993) behaviors, which may be used to monitor the integrity of GABAergic neurons.

In addition to DAergic and GABAergic neurons, neurodegeneration has also been studied in cholinergic neurons. In *C. elegans*, ACh is the major excitatory neurotransmitter, which directly or indirectly regulates locomotion (crawling and swimming) (Mullen et al., 2007), egg laying (Bany et al., 2003), pharyngeal pumping (McKay et al., 2004), defecation cycling (Thomas, 1990), and male mating (Liu and Sternberg, 1995). Gain-of-function nicotinic ACh receptor *acr-2* (Barbagallo et al., 2010) and selenium (Se) (Estevez et al., 2012) are both able to induce cholinergic motor neuron degeneration and paralysis in worms.

Another neurotransmitter, serotonin (5-HT), is synthesized in eight types of neurons and regulates locomotion, defecation, egg laying, male mating and pharyngeal pumping in worms (Mendel et al., 1995; Segalat et al., 1995; Weinshenker et al., 1995; Niacaris and Avery, 2003). The basal slowing response and egg laying assay have been well-characterized in studies on 5-HT signaling. Mutants for 5-HT enzymes, *bas-1* and *cat-4* (both involved in 5-HT and DA biosynthesis), have been used to analyze serotonin-mediated behaviors (Loer and Kenyon, 1993; Waggoner et al., 1998; Sawin et al., 2000). The locomotor behaviors have been used most often for analysis of neurodegeneration and application of exogenous 5-HT inhibits locomotion, but stimulates egg-laying and pharyngeal pumping (Horvitz et al., 1982; Segalat et al., 1995; Weinshenker et al., 1995; Sawin et al., 2000; Rogers et al., 2001; Niacaris and Avery, 2003). In many of the worm behavioral assays it is difficult to determine the effects of DA vs. 5-HT. Degeneration of other types of neurons induced by metal exposure is less studied in worms, thus not discussed here.

Finally, glutamate is important in synaptic transmission, plasticity and disease, with excitotoxicity, inflammation, oxidative stress, and mitochondrial dysfunction implicated in ALS. Excitatory and inhibitory ionotropic glutamate receptors (iGluRs) mediate some behavior and approximately 10 putative iGluR subunits are expressed in *C. elegans (glr 1-8 and nmr 1-2)* (Brockie et al., 2001). Six of the subunits, including *glr-1*, *glr-2*, *glr-4*, *glr-5*, *nmr-1*, and *nmr-2*, are expressed in many of the *C. elegans* interneurons. GLR-1 in particular has been linked to mediating the avoidance of hyperosmotic stimuli (Mellem et al., 2002), control of forward and backward movement (Brockie et al., 2001), foraging behavior (Hills et al., 2004), and long-term memory (Rose et al., 2003). There is also conserved sequence and function of the vesicular glutamate transporter (*eat-4*) (Lee et al., 1999), and other glutamate transporters (*glt-1; glt-3-7*) (Mano et al., 2007).

In summary, *C. elegans* provides a valuable research tool to study the mechanisms of metal induced human neurological disorders by permitting the visualization of different types of neurons and assessment of their function by florescence labeled cell bodies and functional behavior assays, respectively.

## Techniques for studying neurodegeneration in *C. elegans*

Conserved neurotransmitter biology and high homology with mammalian systems make *C. elegans* a unique system for studies on neurodegenerative disease. The processes of synaptic release, trafficking and formation of neurotransmitters are also conserved. Methods to study neurotoxicologic endpoints have included examining the morphology, behaviors, and changes in gene expression and neurotransmitters.

### Tools for assessing neuronal structure

#### Fluorescently tagged neurons and microscopy

Contributing to the investigative value of this species is the transparency of the worm, allowing for *in vivo* visualization of fluorescently labeled neurons. Cell bodies and individual processes can be visualized *in vivo* in the worm. Morphological changes, characterized by puncta, blebs, neuronal absence or shrinkage, presence of vacuoles, dorsal or ventral cord gaps, loss of cell bodies or strand breaks in neuronal processes can be used as indicators of neurodegeneration (Nass et al., 2002; Martinez-Finley and Aschner, 2011). GFP has frequently been employed as a reporter construct to visualize specific neurons and synapses in *C. elegans*. DAergic neurons have been tagged for visualization most frequently through the *dat-1*::GFP reporter (Helmcke et al., 2010; VanDuyn et al., 2010), serotonergic neurons through *tph-1*::GFP (Sze et al., 2000; Nass et al., 2002), GABAergic neurons through *unc-25*::GFP (Cinar et al., 2005), and cholinergic neurons through *unc-1*::GFP (Winnier et al., 1999; Nass et al., 2002), and glutamatergic neuron through *eat-4*::GFP (Lee et al., 1999; Earls et al., 2010). Reporter gene fusions also allow for visualization of neuronal morphology and protein expression patterns. Visualization of the neurons is useful, but it is important to correlate the structural damage with cellular and molecular changes as quenching of GFP fluorescence is a possibility and can result in false data interpretation. Both confocal and standard fluorescence microscopy have been used to visualize fluorescently tagged neurons. Electron microscopy has also been used historically to measure structural integrity and fluorescence-activated cell sorting (FACS) has been utilized for purification.

### Tools for assessing neuronal function

#### Behavioral assays

*C. elegans* behavior is used as a reliable and sensitive measure of the function of neurons as the behaviors are sensitive to underlying cellular and molecular alterations. There are a large variety of behavioral tests in *C. elegans* to assay neurodegeneration. There are nematode equivalents of mammalian DAergic, serotonergic, cholinergic, GABAergic and glutamatergic neurons (see above) and high homology exists between the two systems. A number of behaviors can be assayed to determine the function of the cholinergic and glutamatergic neurons, but these behaviors have not been extensively studied in regards to metals and neurodegeneration, and therefore are not included in this review [for a review see (WormBase, 2013)]. Many of the assays listed below have been designed to assess DA, 5-HT and GABA subtypes.

#### Basal slowing response

Bacterial mechanosensation induces the DA-mediated slowing of locomotion in the presence of food (bacteria), and can be measured by counting the number of body bends per 20-s interval (Sawin et al., 2000). Locomotor rates are compared between the well-fed worms placed on plates of food vs. those placed on plates without food and the measurement is referred to as the change (Δ) in body bends over a set period of time. A lower value represents less slowing on food and therefore deficits in DAergic function as worms deficient in DA do not slow their locomotion in the presence of food. The *cat-2* strain is tyrosine hydroxylase (TH)-deficient and therefore defective in bacterial mechanosensation, making it a positive control (Sawin et al., 2000). Proper controls for the assay include well-fed nematodes and consistency in the size and thickness of the bacterial lawn in all plates used. *Bas-1* and *cat-4* mutants also do not slow in response to the bacterial lawn (Sawin et al., 2000). Application of exogenous serotonin can rescue the behavior in *bas-1* and *cat-4* mutants. In this assay the key to parsing out 5-HT effects from DA effects is the state of the worms; the 5-HT version of this task requires starved worms, while the DA version requires that the worms be well-fed.

#### Ethanol preference

Ethanol preference in *C. elegans* requires DA and serotonin. This has been shown using *cat2* and *tph-1* mutants as these worms do not show a normal wildtype response in the ethanol preference task (Lee et al., 2009). In this assay young adult worms are preincubated on a seeded control or ethanol plate. After an incubation period the worms are removed from the preincubation plates and placed on assay plates. Assay plates are divided into quadrants, with two quadrants laced with ethanol and the other without. Worms are placed at an origin point of an assay plate and allowed to move for 30 min at which point they are scored for their quadrant of preference. A preference index (PI) is calculated as [(number of worms in ethanol quadrants) − (number of worms in control quadrants)]/Total number of worms tested (Lee et al., 2009). Worms with functional DA and 5-HT develop ethanol preference whereas mutant worms do not.

#### Area-restricted searching (ARS)

This task capitalizes on the fact that worms must have functional DA in order to search for food. Wildtype worms that have consumed and exhausted the immediate food source will expand their search to surrounding areas. This behavior is measured by time-dependent reduction in turning frequency after the last food encounter. After a short time if the worm cannot find more food in the immediate area, the frequency of high-angled turns is reduced and the animal begins to explore more distant areas. Functional DA is required for ARS as ablation of dopaminergic neurons eliminates ARS behavior and preincubation with DA restores the ARS behavior in worms with defective DA (Hills et al., 2004). The glutamatergic neurons also participate in this behavior as glutamate receptor mutants exhibit problems with the ARS behavior (Hills et al., 2004).

#### Habituation task/tap withdrawal response

Functional DA allows worms to alter their behavior in the tapping task. Nematodes respond to a mechanical stimulus (plate tapping) by accelerating their forward locomotion rate or by swimming backwards. Repeatedly tapping of the plate results in worms habituating to the stimulus and decreasing the frequency of their reversals. What is measured is the time that it takes to habituate to the tapping rather than whether or not the worm responds to the tapping. All worm strains tested in this task are able to reduce their reversal rates but the distinguishing factor of whether DAergic neurons are functional or dysfunctional is the lag time in response. Intertapping interval is also an important consideration in this assay. *cat-2* mutant worms habituate to tap faster than wildtype worms and this response can be rescued by pre-exposing mutants to exogenous DA (Groves and Thompson, 1970; Broster and Rankin, 1994; Rose and Rankin, 2001; Sanyal et al., 2004).

#### Pharyngeal pumping and thrashing behaviors

Pharyngeal pumping and thrashing rates are controlled by various mechanisms in the nematode. The pharynx is responsible for feeding via rhythmic contractions. Age-related decline in pharyngeal pumping rate has been described in *C. elegans* and is correlated with a decline in survival probability and body movement decline. Pumps per minute are manually counted using a dissecting microscope to assess pharyngeal pumping rate. The maximum pumping rate of the pharynx is ~300 pumps per minute (in adults that are at least 2 days old). Thrashing behavior is a common test in *C. elegans* for measuring motility. Thrashing behavior is assessed by videotaping nematodes that are placed in ~10 μL of water on a transparent plate with shallow, concave wells. A thrash is a change in direction in midbody bending. A body bend is counted as a change in the direction of the posterior bulb of the pharynx. There are multiple programs used to assess this behavior including Worm Tracker, Nemo, Parallel Worm Tracker, OptoTracker, Multimodal illumination, and tracking system, CoLBeRT, many of which use MATLAB, JAVA, or LabVIEW software to quantify movement (WormBase, 2013). This assay, along with visualization of the neurons, has been used to assess GABAergic function after lead or mercury exposure (Helmcke et al., 2009; Xing et al., 2009b).

### Measurement of neurotransmitters

The neurotransmitters, DA and serotonin, and neuromodulators, octopamine and tyramine, can all be detected in *C. elegans* extracts by high-performance liquid chromatography (HPLC) (Sulston et al., 1975; Horvitz et al., 1982; Sanyal et al., 2004; Alkema et al., 2005). Thousands of worms (over 30 ul of packed worms) must be used for HPLC analysis and the worms must be washed several times to avoid bacterial contamination (Sulston et al., 1975; Horvitz et al., 1982; Sanyal et al., 2004; Alkema et al., 2005).

### Using *C. elegans* as a model of neurodegeneration: modifying genetics

The *C. elegans* model system has become a popular and innovative tool in advancing mechanistic studies concerning various neurodegenerative processes. The generation of genetic knockouts and knockdowns can be achieved rapidly in nematodes due to their quick life cycle and short lifespan. Classical forward genetics studies involve treating organisms with mutagens to induce DNA mutations followed by isolation of individuals with atypical phenotypes of interest. Reverse genetics allows the opposite strategy by altering a specific gene in order to study its function and the role it may play in various processes and pathways. RNA interference (RNAi) and transgenesis, which can be accomplished using microinjection and gene bombardment techniques are also tools for achieving gene knockdown.

#### Microinjection

This approach uses a microinjection needle to introduce a plasmid carrying the gene of interest into the cytoplasmic core of distal gonad arms in a hermaphrodite worm, allowing the delivery of the plasmid to several germ cell nuclei and the production of extrachromosomal DNA arrays (Ahringer, 2006). The simplest approach to transgenesis typically involves microinjecting a plasmid that contains a cloned 5′ regulatory sequence of the gene of interest fused to a reporter gene whose activity can then be assayed; a 3′ UTR should also be included (Praitis and Maduro, 2011). As the worm is fully transparent, it is usually desired to fuse genes of interest to a fluorescent reporter whose intensity can then be imaged, such as GFP, yellow fluorescent protein (YFP), or m-cherry (Miller et al., 1999). There are also sequences specific for subcellular targeting that can be included in the plasmids, such as a nuclear localization signal (NLS) for nuclear targeting or a mitochondrial targeting sequence (MTS) for mitochondrial localization. Similarly, tissue or cell-specific expression can also be studied using transgenes that are directed by tissue or cell-specific promoters (Praitis and Maduro, 2011). Additionally, it is typical to include a coinjection marker (antibiotic resistance, tissue-specific fluorescent promoter, etc.) to follow the transgene through the transformation process in larvae and subsequent crossing. However, this is not always necessary if the transgene containing the gene of interest includes a marker itself that can be conveniently distinguished from non-transgenic worms (e.g., a bright GFP reporter).

Following microinjection of transgenes, the DNA arrays produced are extrachromsomal and are not fully integrated into the genome (Ahringer, 2006). Without integration, the arrays remain unstable and can be lost through successive generations. Gamma or UV irradiation or exposure to chemical mutagens can induce integration of the arrays into chromosomes. A small population of the transformed nematodes is mutagenized, resulting in several F1 animals that produce the F2 generation that can then be assayed for 100% transmission of the transgene. Integration of the original transgene is vital for any further genetic manipulation of the transformed lines, eventually allowing multiple transgenes to be incorporated into the same strain (Praitis and Maduro, 2011). As with most genetic manipulations, integrated strains should be backcrossed several times in order to remove any mutations introduced into the genome from the integration process.

However, high copies of the transgene in the extrachromosomal array can lead to undesirable expression of the transgene, ranging from increased transgene expression relative to the targeted endogenous gene to gene silencing from tandem repeat sequences present in the array. For this reason, another method of transgenesis uses microparticle bombardment. Biolistic transformation allows for direct insertion of transgenes into the chromosome, especially with a low-copy number that becomes fully integrated. Bombardment is usually done with the DNA-coated gold beads that are then bombarded onto L4 and adult hermaphrodites. This technique revolves around the ability of DNA to form a complex with gold particles in the presence of CaCl_2_, where the DNA itself is protected from degradation by using cationic polyamines (e.g., spermidine) (Isik and Berezikov, 2013). Researchers have also found that this “gene gun” technique not only reproducibly allows for invariable expression levels and patterns that are difficult to obtain with extrachromosomal arrays, but that low-copy transgene expression does not get silenced in the germline (Praitis et al., 2001). Newer techniques to allow single- or low-copy transgene expression without bombardment are in the process of development, including the use of ultraviolent trimethylpsoralen (Kage-Nakadai et al., 2012).

#### RNA interference (RNAi)

This methodology involves the exogenous introduction of double-stranded RNA (dsRNA) that is complementary to a specific sequence of the gene of interest into the model organism, resulting in the activation of an endogenous cellular pathway that causes significantly decreased expression of that gene. This technique is advantageous in the *C. elegans* system and is dependent on RNA-dependent RNA polymerases (RdRPs) (Simmer et al., 2002); unlike mammals, RNAi is actually heritable in nematodes, as the systemic gene knockdown can persist in the progeny.

RNAi effect can be achieved in three ways: (1) by microinjection of a dsRNA sequence complementary to the gene of interest into the body cavity, including the gonad and intestine, (2) by soaking worms in a dsRNA-containing solution, and (3) by feeding worms with *E. coli* expressing the dsRNA of interest (Tabara et al., 1998).

The unique, highly systemic nature of RNAi in *C. elegans* is thought to occur due to a rapid transport of dsRNA from cell to cell through the SID-1 channel. This transmembrane protein allows for passive, bidirectional transport to cells that do not initially receive the dsRNA delivery, and is selectively gated by the presence of dsRNA (Shih and Hunter, 2011). Wildtype N2 strains are sufficient in producing knockdowns using RNAi; however, some RNAi-hypersensitive strains can be necessary to increase the knockdown strength and phenotype penetrance. These strains include *rrf-3*, *eri-1*, and *lin-15B*. *rrf-3* is a *C. elegans* RNA-dependent RNA polymerase (Simmer et al., 2002), while *eri-1* (enhanced RNAi) is a 3′-5′ exoribonuclease that negatively regulates RNAi (Kennedy et al., 2004) and *lin-15B* is important in cell differentiation and negatively regulates vulval development, as well as RNAi (Wang et al., 2005). There are currently two commercially available RNAi feeding libraries. The Ahringer lab library contains about 16,757 clones that were constructed from cloning gene-specific genomic sequences between two T7 promoters. The Vidal lab library contains 11,511 clones and was made by the Gateway cloning system that clones full-length open reading frames (ORF) templates into double T7 vectors. Together, these libraries cover approximately 94% of the *C. elegans* genome (Ahringer, 2006; Antoshechkin and Sternberg, 2007).

#### Gene knockout

In comparison to RNAi-mediated gene knockdown, knockout in *C. elegans* was made simple by the development of the MosDel system, or Mos-mediated deletion. A plasmid containing Mos1 is delivered to a DNA strand that is next to the target gene, causing a break in that strand. Upon induction of cellular DNA damage repair mechanisms, a DNA template that lacks the targeted gene of interest is introduced and matches the sequence of the broken DNA strand (Frokjaer-Jensen et al., 2010). Thus, this technique tricks the cell into knocking out the gene by repairing the original double-strand break with a DNA template that does not contain the gene of interest. Large-scale projects have aimed to utilize the MosDel system to create large libraries of transposon-tagged alleles (Bazopoulou and Tavernarakis, 2009). Prior to the development of this method, knockouts were primarily generated using random chemical mutagenesis (i.e., EMS mutagenesis) to create loss-of-function mutants that were then screened using gene-specific primers for random deleted regions in targeted genes. However, with this technique there is the possibility of random background mutations and it is not as specific as the MosDEL system. The commercial availability of deletion mutants is possible through the *C. elegans* Gene Knockout Consortium (GKC) and the National BioResource Project of Japan (NBRP), both of whom send their isolated deletion mutants to the *Caenorhabditis* Genetics Center (CGC) for distribution (Antoshechkin and Sternberg, 2007).

### Mechanisms of neurodegeneration induced by metal

#### Aluminum (Al)

Al is one of the most abundant metal elements in the Earth's crust and exposure to bioavailable Al may be significant owing to its ubiquitous presence in soil and fertilizers, cookware, and pharmaceutical and cosmetic preparations (Verstraeten et al., 2008). Although the physiological requirement for Al has yet to be defined, it has been posited that Al may have an etiopathogenic role in neurodegenerative diseases (Halatek et al., 2005). Studies with Al in the form of AlCl_3_, Al(NO_3_)_3_, or Al_2_(SO_4_)_3_ identified phenotypic abnormalities, including toxic effects on lifespan, body size, development, reproduction, locomotion, behavioral plasticity, and memory in the worm (Wang et al., 2009; Page et al., 2012). Additionally, Page et al. showed changes in elemental composition of whole worms exposed to Al, noting a significant increase in multiple metals and ensuing oxidative stress (Ba, Fe, Cr, and Cu), hypothesizing that altered levels of these elements contributed to the aforementioned phenotypes seen in chronic Al toxicity (Page et al., 2012).

VanDuyn et al. addressed molecular attributes of Al-induced DA neurodegeneration in *C. elegans* (VanDuyn et al., 2013). A brief exposure to Al decreased mitochondrial membrane potential and cellular ATP levels and led to DAergic neurodegeneration, which was dependent upon SMF-3, a homolog to the human divalent metal transporter (DMT1). Al exposure significantly decreased SMF-3 protein levels and exacerbated DAergic neurodegeneration in the presence of human PD-associated protein α-synuclein, Nrf2/SKN-1 and Apaf1/CED-4. Deletion of SMF-3 conferred resistance to Al due to sequestration of Al in an intracellular compartment (VanDuyn et al., 2013). Neuroprotection was also reported by Ye et al. showing that a treatment with trace vitamin E could ameliorate memory deficits in Al exposed worms (Ye et al., 2008).

The acute and chronic effects of Al_2_O_3_ nanoparticles (NPs) in *C. elegans* have been recently addressed (Yu et al., 2011; Li et al., 2012). Acute toxicity was associated with increased lethality and altered growth, reproduction and stress responses, whereas chronic toxicity led to increased oxidative stress. Li et al. noted decreased locomotion behaviors in nematodes chronically exposed to Al_2_O_3_-nanoparticles (NPs) concomitant with increased reactive oxygen species (ROS) generation and disruption of ROS defense mechanisms, secondary to the suppression of Mn-SODs (Li et al., 2012).

#### Copper (Cu)

The essential trace element Cu serves as a cofactor in many critical biological processes such as in iron (Fe) homeostasis (ceruloplasmin), catecholamine biosynthesis (tyrosinase, dopamine-b-hydroxylase), oxidative phosphorylation (cytochrome c oxidase), and oxidative stress protection (superoxide dismutase) (Arredondo and Nunez, 2005). Therefore, Cu deficiency or overload may result in multiple pathologies, including irreversible CNS damage (Prohaska, 2000; Taly et al., 2007; Diaz-Veliz et al., 2008). In *C. elegans*, Cu deficiency led to decreased Cu/Zn superoxide dismutase (SOD), reducing defenses against oxidative stress (Yang et al., 1998). Exposure to excess CuSO_4_ induced detrimental effects on brood size and life span, an increase in generation time and impaired development (Harada et al., 2007; Calafato et al., 2008). PD worms expressing α-synuclein or lacking parkin do not show increased sensitivity to Cu toxicity (Ved et al., 2005). In the context of AD, Cu has been shown to increases amyloid deposits and Aß oligomer aggregation, and decrease the amount of soluble Aß oligomer (Rebolledo et al., 2011). The increased Aß aggregation is associated with improvement in behavioral deficits and synaptic function. This protective effect is not attributable to the activation of the SKN-1/NRF2 phase II detoxification pathway (Rebolledo et al., 2011). Luo et al. noted that high Cu concentrations significantly increased the paralysis rate of the Aß (1–42) transgenic worms, whereas lower Cu concentrations decreased paralysis rate. ROS were identified to be responsible for the paralysis and the ROS induced by Aß (1–42) and Cu was mediated through *sod-1*, *prdx-2*, *skn-1*, *hsp-60*, and *hsp-16.2* genes (Luo et al., 2011). The amyloid precursor protein (APP) of AD has a copper-binding domain (CuBD) located in the N-terminal cysteine-rich region that can strongly bind Cu (II) and reduce it to Cu (I). The CuBD of *C. elegans*, APL-1, protected against Cu-mediated lipid peroxidation and neurotoxicity, therefore the CuBD of APP is predicted to play a role in detoxification of neuronal Cu (White et al., 2002; Cerpa et al., 2004).

#### Cadmium (Cd)

Cd is an environmental pollutant that has been classified as a category 1 human carcinogen (IARC, 1993). Cd exposure is associated with teratogenic and mutagenic responses (WHO, 1996). The major routes of human exposure include diet and cigarette smoke (EFSA, 2009). In *C. elegans*, Cd has been shown to alter behavior. It also resulted in decreased growth, life span and reproduction and affected feeding and movement (Popham and Webster, 1979; Boyd et al., 2010; Hoss et al., 2011; Hsu et al., 2012). Cd exposure was also shown to cause GABAergic neurodegeneration in worms. At low Cd concentration neuronal loss was observed, while at high Cd concentration axonal degeneration and neuronal loss, as well as reduced size of AVL, RMEs and RIS neurons was noted in fluorescently labeled GABAergic neurons (Du and Wang, 2009).

#### Iron (Fe)

The essential trace element Fe exists abundantly in the environment and is present in various enzymes and proteins. It has a central role in many essential cellular processes such as DNA synthesis, mitochondrial respiration, oxygen transport, and neurotransmitter synthesis (Cairo et al., 2002). Fe homeostasis has to be maintained within a small range because a dysregulation caused by Fe deficiency or overload leads to hematological, neurodegenerative and metabolic diseases (Dusek et al., 2012; Tandara and Salamunic, 2012). Genes involved in Fe and energy homeostasis in vertebrates are conserved in the nematode. These include aconitase, ferritin, divalent metal transporter-1 (DMT-1), frataxin, and Fe sulfur cluster assembly proteins. The Fe regulating protein-1 (IRP-1) homolog (ACO-1) of *C. elegans* has aconitase activity and is post-translationally regulated by Fe. Although ACO-1 is predicted to be the IRP-1 homolog in *C. elegans*, it fails to bind to the ferritin mRNAs and no conserved Fe responsive elements have been found on ferritin mRNAs in *C. elegans* (Gourley et al., 2003).

In contrast, Kim et al. reported that *aco-1* and *ftn-1* expression levels are regulated by Fe. Both aco-1 and *ftn-1* gene expression is inversely correlated. *ftn-1* and *ftn-2* are encoding ferritins in *C. elegans* (Kim et al., 2004). In mutant animals lacking ACO-1 and FTN-1, reduced lifespan has been observed, indicating that *aco-1* and *ftn-1* are important for regulating Fe homeostasis. *daf-16* mutants show decreased lifespan upon Fe treatment, suggesting that DAF-16 signaling might be also involved in Fe homeostasis (Kim et al., 2004). HIF-1 (hypoxia-inducible factor-1) is a negative regulator of ferritin transcription, inhibiting *ftn-1* and *ftn-2* transcription during Fe deficiency. Furthermore, the activation of the Fe uptake gene smf-3 (a homolog of DMT-1) by HIF-1 during Fe-deficiency (Romney et al., 2011) provides a mechanism to maintain sufficient Fe stock for growth and survival when Fe is limited in *C. elegans*.

Fe overload in worms causes phenotypic and behavioral defects as well as alteration the resistance to oxidative stress, characterized by reduced lifespan, body size, generation time, brood size, head thrash and body bend frequencies, as well as chemotaxis plasticity (Hu et al., 2008; Valentini et al., 2012). Several of these defects (body bend frequency and life span) were transferred from Fe-exposed *C. elegans* to their progeny (Hu et al., 2008). The adverse effects of Fe on locomotive behavior suggest that Fe might be involved in disruption of synaptic function between neurons and muscle cells. In *C. elegans* models of Aß toxicity, Fe was shown to possess high affinity for Aß. Aß accumulation in the Aß-expressing strain CL2006 resulted in Fe homeostasis disruption. In addition to increasing Fe content, Aß has also been shown to increase ROS generation (Wan et al., 2011).

#### Lead (Pb)

Lead is a pervasive environmental neurotoxicant; it is particularly toxic to developmental brains, causing long-term detrimental effects on learning, memory and behavior in children (Neal and Guilarte, 2010). Pb exposed *C. elegans* show severe abnormalities in their lifespan, development, locomotion, learning and memory behaviors (Ye et al., 2008; Zhang et al., 2010). Younger larva (L1, L2, L3) nematodes exhibited increased lethality and reproductive toxicity (brood size, generation time) compared to L4 stage or adult nematodes (Guo et al., 2009; Xing et al., 2009a). L3 larvae showed higher sensitivity in transgenerational behavioral and growth effects than older life stages (Yu et al., 2013). Neurodegeneration, as shown by neuronal loss and dorsal/ventral cord gaps is more pronounced in L1 larvae compared to older nematodes (Du and Wang, 2009; Xing et al., 2009b).

Pb exposure decreases thermotaxis behaviors in adult *C. elegans*. This behavior is mainly controlled by the AFD sensory neurons and it has been shown that Pb caused severe deficits in the structural properties of these neurons. Pre-treatment with antioxidants inhibited ROS elevation and mitochondrial dysfunction caused by Pb and prevented the induction of oxidative damage, severe deficits in thermotaxis and damage to the AFD sensory neurons (Wu et al., 2012). Vitamin E and mild UV radiation or a pre-treatment with heat shock increase the resistance of nematodes to Pb toxicity, ameliorating the effects on locomotion behaviors, stress response and memory deficits (Ye et al., 2008; Wang et al., 2010; Ye et al., 2010).

#### Methylmercury (MeHg)

Mercury (Hg) and methylmercury (MeHg) are of greatest concern as MeHg is an ubiquitous environmental contaminant (Sanfeliu et al., 2003) and a major source of human exposure is associated with the consumption of seafood (Fitzgerald and Clarkson, 1991). McElwee et al. compared the toxicity of HgCl_2_ and MeHgCl, noting the latter is significantly more toxic than HgCl_2_ when assessing feeding, movement and reproduction, and in its ability to increase the steady-state levels of stress response genes (McElwee and Freedman, 2011). Helmcke et al. showed that while lethality, pharyngeal, pumping, growth and development were affected in *C. elegans*, brood size, lifespan, trashing rate, and nervous system morphology were largely unaffected in response to MeHg exposure. Concerning Hg uptake in *C. elegans*, its concentrations are as high as those found in mammalian systems (Helmcke et al., 2009; Helmcke and Aschner, 2010). It is also confirmed that the involvement of oxidative stress in MeHg toxicity is through alterations in GSH levels, increasing expression of HSP and glutathione-S-transferase (Helmcke and Aschner, 2010). In mammals, Nrf-2 regulates oxidative stress response. The worm Nrf-2 homolog, SKN-1, is also expressed in *C. elegans* DA neurons and a reduction in *skn-1* gene expression increases MeHg-induced DAergic neurodegeneration (VanDuyn et al., 2010).

#### Manganese (Mn)

The naturally abundant micronutrient Mn is an essential trace element and is of crucial importance as co-factor for a wide variety of enzymes, including arginase, pyruvate carboxylase, the antioxidant enzyme SOD, as well as enzymes involved in neurotransmitter synthesis and metabolism (Santamaria and Sulsky, 2010). An imbalance in Mn homeostasis caused by either Mn deficiency or overload is well known to cause severe CNS dysfunction, whereas Mn deficiency is because of its ubiquitous presence in food extremely rare. Therefore, several mechanisms are involved in maintaining the Mn homeostasis. The NRAMPs/divalent metal transporters play distinct roles in Mn transport and the identified *C. elegans* homologs (SMF-1, SMF-2, and SMF-3) have been characterized with corresponding roles in Mn homeostasis and sensitivity (Au et al., 2009; Bandyopadhyay et al., 2009; Settivari et al., 2009). The three SMFs differ in localization and function, and SFM-3 has to be shown as the major Mn transporter. A deletion of the *smf-1* or *smf-3* increased Mn tolerance against Mn-induced DAergic neurodegeneration with *smf-3* as the most resistant mutant, whereas the deletion of *smf-2* increases Mn sensitivity indicating the protective role of *smf-2* against Mn (Au et al., 2009). The DA reuptake transporter, DAT-1, has also been associated in Mn toxicity and dysfunction sensitizes the worm to Mn neurotoxicity (Chen et al., 2006; Benedetto et al., 2010). In *C. elegans* PMR1, a P-type Ca^2+/^Mn^2+^ ATPase is involved in the regulation of Ca and Mn ions and PMR-1 knockdowns can render resistance to stresses, such as oxidative stress (Cho et al., 2005; Bandyopadhyay et al., 2009). McColl et al. provide the first quantitative subcellular visualization of endogenous Mn concentrations in *C. elegans* identifying Mn enriched within specific cell types, especially intestinal cells (McColl et al., 2012).

Concerning Mn toxicity in *C. elegans*, it has been investigated that the anti- or pro-oxidative role for Mn is dependent on the Mn-concentration worms are exposed to (Lin et al., 2006). In case of Mn overload accelerated development, an increase in total fertility, reduced body and brood size and life span was observed (Lin et al., 2006; Xiao et al., 2009). Mn toxicity in *C. elegans* has been associated with increased ROS formation and glutathione production, head mitochondria membrane potential and DA neuronal death (Settivari et al., 2009; Benedetto et al., 2010). Benedetto et al. found that Mn-induced oxidative stress was dependent on the extracellular levels of DA, which was confirmed by using strains lacking functional DA receptors (DOP-1, 2, 3) and the DA transporter (DAT-1) that regulate extracellular DA, and strains lacking vesicular monoamine transporter 2 (CAT-1 in worms) and tyrosine hydroxylase (CAT-2 in worms) that regulate intracellular DA levels. Decreased extracellular DA levels are protective against Mn toxicity (Benedetto et al., 2010). They identified NADPH dual-oxidase, BLI-3, potentiates the formation of RONS from DA-derived species obtained by the Mn (II) and extracellular DA exposure, while the bli-3 mutant a hyper-resistant phenotype of Mn toxicity showed which could be a potential therapeutic target against Mn toxicity (Benedetto et al., 2010). Consistent with the oxidative stress associated with Mn exposure, SKN-1 (antioxidant transcription factor contributing to protection against ROS; homolog of mammalian Nrf2) overexpression or nuclear relocalization of SKN-1 protects from Mn neurotoxicity indicating a beneficial strategies in limiting Mn toxicity (Benedetto et al., 2010). Prominent theories on neurodegeneration implicate further protein aggregation, including α-synuclein. Recently suppression of α-synuclein-induced toxicity has been demonstrated by expression of PARK9 encoding a putative P-type transmembrane ATPase (ATP13A2) protein (Gitler et al., 2009).

#### Zinc (Zn)

Zn is one of the most prevalent essential elements. It is a cofactor in many enzymes and transcription factors involved in several cellular processes and cellular signaling pathways (Shuttleworth and Weiss, 2011). Zinc deficiency as well as excess causes a wide spectrum of defects in multiple organ systems. Zn's role in memory, learning, neurogenesis, processes related to brain aging and neurological diseases have been extensively reviewed (Szewczyk et al., 2011).

In *C. elegans*, families of the CDFs (cation diffusion facilitators), ZIPs (Zrt- and Irt-like proteins) and MTs (metallothioneins) are involved in Zn metabolism. Deletion of MT-1 and MT-2 results in increased Zn accumulation, whilst *mtl-1* knockout worms show heightened sensitivity to increased Zn level (Leszczyszyn et al., 2011). *cdf-1* and *sur-7* are both members of the CDF family, and mutations in either gene result in increased sensitivity to Zn, indicating that both genes are necessary for Zn tolerance. However, *sur-7* mutants were more tolerant than a *cdf-1* mutant to increased Zn concentrations, suggesting functional differences between these two proteins (Yoder et al., 2004). In addition, mutations of haly-1, which encodes an enzyme converting histidine to uric acid, cause elevated levels of histidine and protect against Zn toxicity (Murphy et al., 2011). A loss-of-function study of *cdf-1*, *cdf-2*, *sur-7*, and *haly-1* showed that *haly-1* promotes Zn resistance in the genetic backgrounds characterized by Zn sensitivity and functions in parallel to *cdf* genes in modulating Zn sensitivity. Zn exposure in *C. elegans* results in multiple biological defects affecting life span, reproduction, locomotion behavior (head trash, body bend) and chemotaxis plasticity (Wang et al., 2007). The phenotypic and behavioral toxicities could be transferred from Zn-exposed nematodes to their progeny (Yoder et al., 2004; Wang et al., 2007). While defects such as life span, generation time, brood size, and chemotaxis plasticity could be partially rescued in the progeny, no rescue phenomena could be observed for body sizes and the locomotion behaviors. In addition, it has been reported that the pesticide Mn/Zn-ethylene-bis-dithiocarbamate promotes neurodegeneration in DAergic and GABAergic neurons (Guven et al., 1999; Negga et al., 2011, 2012; Harrison Brody et al., 2013).

## Conclusion

*C. elegans* is a valuable model to study human neurological diseases due to conserved genome, low culture cost, short generation cycle, transparent tissue and simple nervous system, together with well-developed tools. A solid number of nematode models related to human neurological disorders have already been developed, including AD (Miyasaka et al., 2005; Brandt et al., 2009), Parkinson's disease (Cao et al., 2005; Hamamichi et al., 2008), Huntington's disease (Parker et al., 2001; Caldwell et al., 2003), ALS (Vaccaro et al., 2012), and early-onset torsion dystonia (Cao et al., 2005; Chen et al., 2010). These disease models facilitate the study of metal-induced neurological toxicity and many novel discoveries have already been made in *C. elegans* to uncover the mechanisms of metals related human neurological disorders. Despite of these advantages, limitations of the nematode system need to be considered. For example, some genes and signaling pathways inherent to mammals (such as α-synuclein and Aβ discussed above) are missing in this model. In sum, *C. elegans* provide a valuable platform for exploratory study of human neurological diseases by providing mechanistic information on processes inherent to neurodegenerative disorders.

### Conflict of interest statement

The authors declare that the research was conducted in the absence of any commercial or financial relationships that could be construed as a potential conflict of interest.
